# Remote Monitoring of CIEDs—For Both Safety, Economy and Convenience?

**DOI:** 10.3390/ijerph19010312

**Published:** 2021-12-28

**Authors:** Knut Tore Lappegård, Frode Moe

**Affiliations:** 1 Department of Medicine, Nordland Hospital, N-8092 Bodo, Norway; Frode.Moe@nordlandssykehuset.no; 2 Department of Clinical Medicine, UiT The Arctic University of Norway, N-9037 Tromso, Norway

**Keywords:** pacemaker, cardiac implantable electronic device, home monitoring, remote monitoring, telemedicine, e-health, health technology assessment

## Abstract

Cardiac implantable electronic devices such as pacemakers and defibrillators are increasingly monitored by systems transmitting information directly from the patient to the hospital. This may increase safety and patient satisfaction and also under certain circumstances represent an economic advantage. The review summarizes some of the recent research in the field of remote monitoring of cardiac devices.

## 1. Introduction

Since the first implant of a cardiac pacemaker in October 1958 [1,2], the use of cardiac implantable electronic devices (CIEDs) has expanded dramatically, with between one and two million annual implants installed worldwide [3]. In developed countries, implantation rate ranges between 500 and 1500 per million inhabitants per year. A detailed description of implantation rates in European countries can be found in the EHRA White Book [4]. CIEDs are a group of devices which includes transvenous and intracardiac pacemakers (PMs) for treatment of bradycardia, transvenous and subcutaneous defibrillators (ICDs) for treatment of tachycardia and prevention of sudden cardiac death, resynchronization devices (CRTs) for treatment of heart failure, and implantable loop recorders (ILRs) for monitoring the heart rate. The devices are technically highly advanced, and the patients require regular follow-ups. The follow-ups include, but are not limited to, control of battery status, lead integrity and pacing thresholds (when applicable), and occurrence of arrhythmias. The frequency of follow-ups may vary from country to country, and also between the various device types, but one or two annual visits to the hospital has been a common practice. Recent development has made it possible to perform such follow-ups using remote telemonitoring, and this may, under certain circumstances, provide improved safety and patient satisfaction, as well as reduction of costs. According to several organizations, remote device management is *recommended* for certain CIED patients and *should be considered* for all [5,6]. However, regular hospital follow-up visits are still the routine for the majority of patients [7].

**Figure 1:**
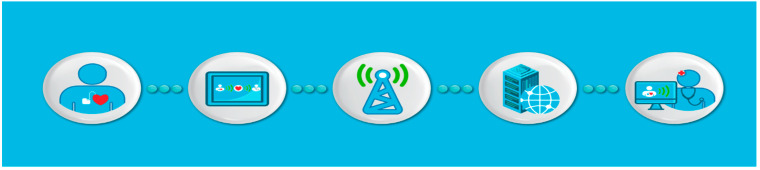
Schematic drawing of the circuit consisting of patient with CIED, transmitter, server, and hospital computer and staff.

Figure 1 shows a schematic drawing of a remote follow-up system. Data is transmitted from the CIED to a monitor (transceiver), which the patient usually places in the bedroom or living room. The transceiver communicates with a server through Wi-Fi or GSM and information from the server can then be downloaded through a secure channel to a hospital computer. The clinical personnel (doctors or dedicated nurses/technicians) only have access to the patients belonging to their clinic. There are several mechanisms through which a transmission from the patient can be activated: It can be “on demand”, i.e., initiated by the patient because of experienced symptoms or following a request from the clinic; it can be event-driven, i.e., as a result of a technical issue in the CIED or, e.g., the occurrence of an arrhythmia; or it can be a scheduled transmission taking place on a given date and time. The system can be set up according to patient history as well as the preferences and resources of the clinic. In order to take full advantage of the possibilities in the remote monitoring (RM) system, the organization of the clinic is important [8]. As is discussed later, when and how the information downloaded is processed by the clinic will affect a number of aspects regarding safety, economy, and patient satisfaction.

**Figure 2:**
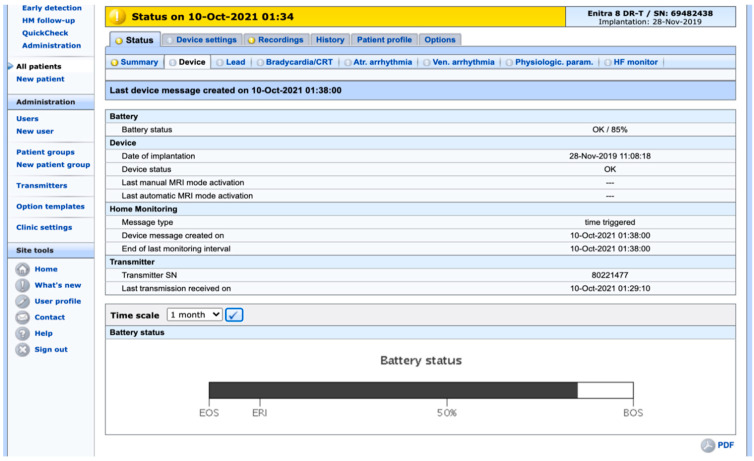
Screenshot from a web page in one of the RM systems (Biotronik Home Monitoring™). A number of pages/folders are available for each patient.

**Figure 3:**
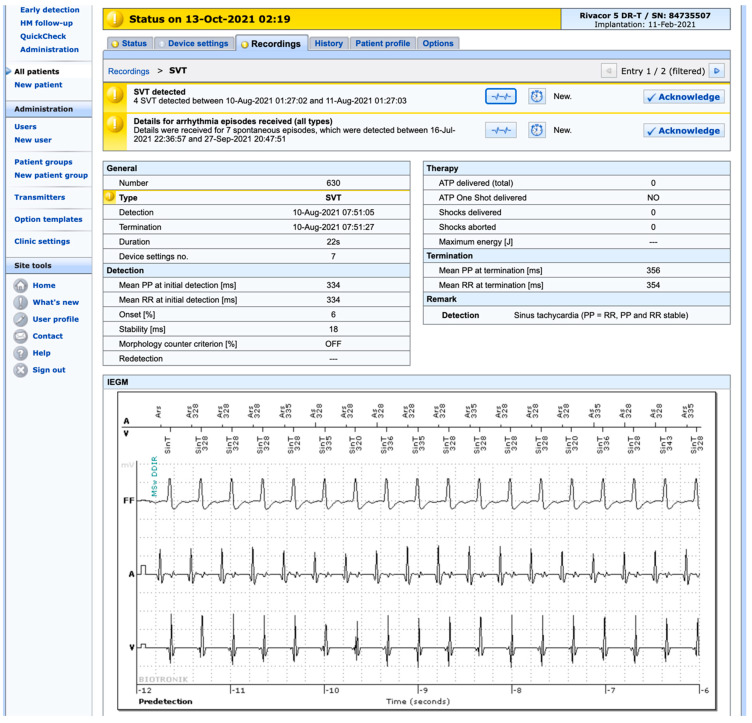
Screenshot from a web page in one of the RM systems (Biotronik Home Monitoring™, Biotronik SE & Co. KG, Berlin, Germany) showing an episode of “yellow alert” involving a supraventricular tachycardia.

The information downloaded will appear on the screen in a format defined by the supplier/vendor of the CIED. Although the web pages differ in layout, for all practical purposes, they contain the same information, such as battery status, function of the lead(s), and occurrence of arrhythmias, as well as a number of other parameters. Figure 2 shows one of several pages from the RM system of a particular vendor and a particular patient, indicating how the information is presented. Figure 3 shows the occurrence of a supraventricular arrhythmia, an event that has prompted a “yellow alert” to the hospital. How the various events regarding device function and clinical status are classified is determined by the clinic when setting up the system for the individual patient. This also applies to how the alerts are conveyed to the clinic. For instance, a serious “red alert” can trigger an immediate text message or phone call to a specific phone, whereas a less-serious “yellow alert” will be sent by email and thus may not be acted upon until the next morning.

A well-designed system for RM is, by now, considered an integral part of the product when purchasing a CIED. The largest vendors of CIEDs worldwide each offer their specific platform for remote monitoring, and usually all the different devices (pacemakers, ICDs, CRTs, and ILRs) of a company are compatible with their RM platform (Table 1).

Two other CIED brands on the international market, Pacetronix and Lepu, currently have no home monitoring system.

From a number of perspectives, remote monitoring of CIEDs may offer obvious advantages when it comes to safety, economy, and patient convenience. However, RM is also associated with challenges and limitations. The paper gives a summary of recent research in this field.

## 2. Safety

Patient safety is the primary objective in the follow-up of CIED patients, whether it is in hospital or through RM. Safety concerns several aspects. One part is related to basic functions of the CIED, such as battery status, lead integrity (including damage to insulation or conductor), and changes in pacing threshold with the risk of loss of capture. Nishii and coworkers have shown that RM can detect early lead failure, contributing to increased safety [9]. Another part relates to the occurrence of significant arrhythmias, detection of which may have therapeutic consequences for the patient such as anticoagulation for atrial fibrillation or antiarrhythmics for ventricular tachycardia. Some CIEDs also have the ability to detect sleep apnea, another clinical situation which may trigger therapeutic intervention. Furthermore, occasionally, a pacemaker manufacturer will issue a recall or a so-called field warning regarding a certain product where intensified follow-up is required or recommended. Chiu and coworkers recently showed that RM is noninferior to conventional in-hospital follow-up of heart failure patients implanted with a resynchronization device [10]. Thus, in a number of settings, RM can be beneficial without compromising patient safety, and in several studies, improved safety has actually been demonstrated [11,12,13,14].

In one study, RM of CIEDs in patients who suffered from COVID-19 identified patterns of heart rate and thoracic impedance consistent with complications before adverse clinical manifestations were present [15]. Similar findings have been confirmed in other trials and case reports [16,17]. Remote monitoring in the setting of a pandemic such as COVID-19 also provides safety for both health care workers and patients, as limited physical contact reduces the risk of virus transmission. Transmitters can also be delivered at home to patients who have previously not been monitored remotely, to limit physical contact in the setting of a pandemic [18].

When a patient is admitted to a hospital to have a CIED implanted, an overnight stay after surgery has been the common practice, with the exception of ILRs which are implanted on an outpatient basis. However, it has recently been shown that RM allows for ambulatory CIED implantation without compromising safety [19], a practice which will also affect economy.

It has been shown that RM of ICDs reduces both the occurrence of inappropriate shocks and time to medical assessment when used in rural areas [20], and in a large Italian trial involving 1650 patients, RM was effective in detecting and managing clinical events with limited use of resources [21]. Thus, remote monitoring may detect changes of clinical importance and lead to changes in follow-up or medication. For instance, the occurrence of atrial high-rate episodes has been associated with an increased risk of ventricular arrhythmias [22], and early detection of this may cause the physician to contact the patient and suggest changes in medication.

In order for RM to be economically attractive (see below), it is important that it is a replacement for, and not an addition to, hospital follow-up. In a trial of >1200 patients, Watanabe and coworkers showed that it is possible to reduce the number of in-house controls without loss of safety [23].

How the clinic responds to alerts from the system is of importance when it comes to safety, and this will also affect parameters such as economy and patient satisfaction. As previously mentioned, the clinic can receive alerts through a phone message or through an e-mail. Depending on the size of the clinic, responding to such alerts can be 24/7/365 or in ordinary working hours [24]. A major issue is that the patient must receive detailed information regarding what service the clinic can offer in order for expectations to be met. Some patients have the impression that RM is equivalent to continuous real-time surveillance of their heart rhythm, and unless such misunderstandings are corrected early, patient dissatisfaction is bound to occur.

In order to obtain maximum advantage of RM, it appears that early enrolment is of importance. Mittal and coworkers showed improved survival in a large cohort enrolled in RM less than 91 days after CIED implant, compared to patients enrolled later [25]. This study was, however, not randomized, introducing a number of possible explanations for the difference between the groups.

In addition to improving safety for the individual patient, RM is a powerful research tool and may thus affect future patient safety. Remote monitoring can deliver information from an enormous number of patients and provide the scientific community with important data regarding arrhythmias, heart failure, sudden cardiac death, and all-cause mortality. This has been described in several studies [26,27,28,29,30] and also discussed in previous reviews [31].

## 3. Economy

Remote monitoring of CIEDs can affect hospital and patient economy in a number of different ways, and although RM has the potential to be economically beneficial for most clinics, the extent of this must be evaluated in the local setting. Device companies charge differently, and how costs are charged, as well as the actual price, will differ between countries. For instance, some companies charge a one-time fee for every patient included in the RM system so that the hospital buys the transmitter, whereas the use is free of charge. Another model is a monthly or yearly fee as long as data is transmitted. For some companies, the transmitter can be reprogrammed and reused if the patient dies; for others, the transmitter is single-use only.

A recent development is the use of Bluetooth technology in the CIED, allowing communication through this to the patient’s smartphone. The smartphone will then serve as the transmitter and eliminates the need for a special, dedicated device for this purpose. This is both an economic improvement, but also convenient for the patient, especially when traveling, as most patients are closely linked to their smartphone and seldom leave the house without it.

Reimbursement of RM expenses is another area where countries may differ widely [32]. In some countries, health authorities will refund the hospital completely, whereas in other countries, RM will just be an extra expense for the clinic. Furthermore, in some countries, the hospital pays for the patient’s travel to the clinic and will thus save large amounts if the patients can be monitored from home, but in other countries, travel expenses are not covered by the clinics, which then lack this economic incitement. Such differences in reimbursement may explain some of the differences between countries when it comes to the use of RM, and also the differences in results between trials trying to calculate the economic benefit of RM. In a recent Canadian study, Kelly and coworkers found that there were different funding policies across different national jurisdictions, and this represented a barrier to uniform strategies [33].

In addition to the direct expenses connected to the RM systems, economic calculations should also include staff workload and travel cost for the patient, as well as for any caregiver and expenses related to absence from work, when applicable. Not all trials have been able to demonstrate reduced costs for the hospital but reducing costs for the patients is also of importance [34].

In addition to costs in connection with follow-up, it has also been shown that RM may facilitate same-day discharge [19,35] and thus reduce cost related to the implantation.

In the United Kingdom, it has been estimated that the cost of RM is neutral after 10 years [36]. Such calculations will, however, differ between countries due to a number of different factors, as mentioned above. In line with this, there are reports that indicate that remote monitoring is economically advantageous [12,37,38,39,40,41,42], whereas others have been unable to confirm this [43,44]. Comparison between trials is difficult, as some have just calculated the hospital costs (staff time, etc.), whereas others have included travel costs, time away from work, etc. Trials also differ in design in the way that some are randomized, some are nonrandomized, and some compare current practice with historical controls. In nonrandomized trials, where participants were placed in a group according to preference, the possibility of introducing bias is relevant, as the younger and healthier patients may prefer RM and also require less physical follow-up. In line with this, it has been shown that patients who prefer RM tend to be more highly educated and employed as compared to those who prefer hospital follow-up [45].

Remote monitoring of CIEDs requires energy, and questions have been raised as to whether this will deplete battery and lead to more frequent CIED replacements. However, studies indicate the opposite, as ventricular pulse amplitude can be kept lower and the battery status monitored more precisely, both facilitating postponed replacement. Improved longevity of close to a year has been reported [46], an increase which will affect cost significantly [47,48].

Equally as important as saving money may be the fact that RM also saves time compared to in-hospital controls [49]. This allows doctors and nurses to spend more time on tasks that require hands-on and personal attendance.

As results regarding economic advantages of RM are conflicting, performing trials and calculations in local and national settings is necessary if reduction of cost is a prerequisite for the authorities to recommend or agree to the introduction of such a system.

## 4. Convenience and Patient Satisfaction

In general, high patient satisfaction with RM has been demonstrated both in young patients as well as in octogenarians [50], and in nonrandomized [45,51] as well as randomized trials [52] of RM. Although satisfaction is high, it is not obvious how this translates into other health-related outcomes. In a randomized trial of heart failure patients with an implanted ICD, patient-reported health outcomes did not differ between the group that had a 3–6-month hospital follow-up vs. the group with RM and annual hospital visit [53]. In another trial, frequent RM follow-ups, as opposed to usual care, did not affect health-related outcomes in a group of heart failure patients [54]. From a patient perspective, RM may give a sense of increased confidence and security, as hospital personnel will monitor device function and contact the patient in the case of technical or medical issues. However, detailed information regarding response time and to what extent the systems are monitored is essential, as some patients have the misunderstanding that their heart rhythm is under real-time surveillance 24/7/365. Whether an increased feeling of confidence can be translated into standard measures of quality of life is, however, uncertain, as results have been conflicting in nonrandomized [55] and randomized trials [42,56,57]. However, patient satisfaction with RM is, in general, high [8,58,59]. Satisfaction among caregivers, relatives, and friends of CIED patients has also been demonstrated for RM [60].

As previously mentioned, RM may facilitate same-day discharge, which, for the younger and healthier proportion of patients, will be considered as an advantage [19,35].

Patient satisfaction is closely linked to how the patient perceives the availability of health care personnel when encountering symptoms. The patient should have updated information on when, and who, to contact in the event of symptoms. Whether this is on a 24/7/365 basis or limited to in-office hours will depend on local routine and the size of the clinic. In a study from the Czech Republic, octogenarians tended to believe that RM was equivalent to around-the-clock surveillance. This misunderstanding was less common among younger patients [50]. That health care providers and patients may have different perceptions and expectations regarding how the RM system works has also been documented elsewhere [61]. Different health care systems between countries will inevitably lead to differences in practice, but differences within countries due to lack of standards has also been demonstrated [33]. Developing uniform, national standards for the use of RM would probably expand the use and improve patient satisfaction.

In the special setting of a pandemic, such as the recent COVID-19, remote monitoring may be particularly beneficial. The CIED patient will be less exposed to infection both due to reduced travel on public transportation and also less interaction with health care professionals in a hospital system. The use of RM has seen a boost in several countries during COVID-19, as shown in several reports [62,63,64,65].

## 5. Pitfalls and Limitations

There are a number of challenges and possible complications associated with remote monitoring of CIEDs. Technical limitation is one of them. In many countries, transceivers now utilize a 4G, 5G, or Wi-Fi connection. However, in other countries, a wired phone line is the most common. Supplying the patient and transceiver with a cell phone adapter may, in some countries, increase the number of transmissions [66]. For patients living in rural and remote areas far from the hospital, cell phone connection might be a problem, even though such patients will profit the most from RM with regard to reduced travel for hospital follow-up.

Furthermore, successful RM requires trained and dedicated personnel. Continuous transfer of large amounts of data from the patients is of no use if nobody is there to receive the data and to act upon it. The availability of the staff does not always meet the expectations of the patients. Some patients believe they are, and expect to be, monitored around the clock, and it is important to inform the patient in detail what to expect when RM is established. The availability and response time will vary from clinic to clinic depending on size, the number of patients on RM, and the number of people involved in monitoring. It is also of great importance that nurses or technicians involved in monitoring are trained to know when to consult the responsible physician if there are events or alarms in the RM system. Thus, how the clinic organizes the RM follow-up is of great importance [24].

Remote monitoring is also associated with legal and ethical considerations. One is the issue of cybersecurity, a topic that has been thoroughly discussed elsewhere [67]. Another issue is the protection of personal and sensitive data. According to the General Data Protection Regulation (GDPR) of the European Union, collection and processing of personal information is subject to a number of demands and requirements. It is important that both the manufacturers of CIEDs and the clinics receiving data from RM have systems that comply with these regulations. The European Society of Cardiology has published a task force report on this subject [68] and it has also been discussed by others [69]. In addition to data safety, there are legal and ethical issues regarding the data once they are received by the hospital. What should be the response time for the hospital if there is a “red alert”? If an alarm regarding premature battery depletion or lead fracture (leading to severe malfunction of the device) is transferred on a Saturday evening or during Christmas, what is the responsibility for the clinic to act on this? Is it reasonable that such alarms are monitored 24/7/365, or is it sufficient if they are detected on the next working day? Will there be legal repercussions if detection is delayed? Will such repercussions be on a personal level, or is the hospital responsible? These questions may have different answers from country to country, but it is again important to emphasize that expectations should be discussed with the patient before discharge from the hospital. How the clinic and personnel responsible are organized will affect all aspects of RM, including safety, economy, and patient satisfaction [8].

A major concern when being responsible for a large number of remotely monitored patients is the enormous amount of data received from each patient. What kind of information should prompt a phone call to the patients with questions about their clinical condition? What kind of findings should lead to changes in medication? This information overflow has recently been described in a report including >26,000 patients on RM [30]. The amount of information increases workload, and may also pose clinical challenges. As an example, the usefulness of anticoagulation for prevention of thromboembolic complications in atrial fibrillation is well documented. However, the studies upon which this practice is based were performed on patients where atrial fibrillation was diagnosed with an electrocardiogram in the hospital, and the arrhythmia had to be of a certain duration. Remote monitoring of CIEDs may now lead to detection of short (frequent or rare) episodes of atrial fibrillation and it is not clear at what time anticoagulation should be recommended. This challenge was demonstrated in a study by Ricci and coworkers, where stroke incidence was lower than expected from an ordinary risk estimate in a group of remotely monitored CIED patients with automatically detected episodes of atrial fibrillation [70]. In line with this, Rovaris and coworkers found only a moderate correlation between ordinary risk factors for stroke and the occurrence of atrial high-rate episode in a cohort of 2410 patients with CIED without previously documented atrial fibrillation [71].

In addition to the challenges mentioned above, rare technical issues have also been reported, such as problems with RM in a patient who had two CIEDs [72].

## 6. Future Possibilities

The use of RM has, until now, solely been a one-way communication of data that is transmitted from the patient to the physician or hospital. A question frequently encountered when caring for these patients is whether it is possible to remotely program their device. If a problem is detected through RM (e.g., loss of capture due to increased pacing threshold) it would be very convenient for the patient if this could be solved without the patient having to travel to the hospital. This is technically feasible and has been carried out [73], and is probably an area where the CIED companies will direct future research and development. However, the practice of remote programming raises a number of new questions, not the least regarding legal responsibilities.

Another possibility is the application of machine learning. The servers of the various CIED companies contain information regarding the heart rhythm of hundreds of thousands, or millions, of patients. Applying machine learning to this information may, for instance, reveal patterns that precede serious events, such as ventricular arrhythmias or sudden cardiac death, with the prospect of designing algorithms for prevention.

## 7. Conclusions

Remote monitoring may represent a major improvement in the follow-up of patients with CIEDs. This is true from the perspectives of both safety, economic and patient satisfaction. However, clinical studies are in some respects conflicting and successful implementation of a remote monitoring system requires careful patient selection and information as well as an in-hospital system with skilled and dedicated personnel.

## Figures and Tables

**Table 1:** Six major CIED companies with their corresponding home monitoring platform. Not all brands are available in every country.

Abbott (Sylmar, CA, USA)	Merlin.Net™
Biotronik (Berlin, Germany)	Home Monitoring™
Boston Scientific (Marlborough, MA, USA)	Latitude™
LivaNova/MicroPort (Paris, France)	Smartview™
Medico (Padova, Italy)	Ermes™
Medtronic (Minneapolis, MN, USA)	CareLink™

## Data Availability

Not applicable.
